# Effects of Birth Weight on Animal Performance, Fattening Traits and Meat Quality of Lambs

**DOI:** 10.3390/ani10122364

**Published:** 2020-12-10

**Authors:** Sonia Andrés, Carmen Valdés, Alba Santos, Javier Mateo, Francisco Javier Giráldez

**Affiliations:** 1 Instituto de Ganadería de Montaña, CSIC-Universidad de León, Finca Marzanas s/n, 24346 Grulleros, Spain; cvals@unileon.es (C.V.); alba.santos@igm.csic.es (A.S.); j.giraldez@eae.csic.es (F.J.G.); 2 Facultad de Veterinaria, Universidad de León, Campus Vegazana s/n, 24071 León, Spain; jmato@unileon.es

**Keywords:** intrauterine growth restriction, fetal programming, feed efficiency, birth weight, intramuscular fat, lamb, developmental origins of health and disease

## Abstract

**Simple Summary:**

It is accepted that a low body weight at birth (<4 kg) might impair postnatal muscle and increase adipose tissue development during the whole life of lambs. Therefore, in the present study, we aimed to evaluate the effect of body weight at birth of lambs on the growth performance, ruminal parameters, digestibility, non-carcass components, carcass traits, physicochemical characteristics and fatty acid profile of meat when slaughtered at 27 kg. Compared to the lambs born with a high weight (>5.5 kg), the lambs born with a low body weight showed a worse animal performance during the fattening period, and increased fat depots with a higher content of saturated fatty acids and a reduced tenderness of meat.

**Abstract:**

Intrauterine growth restriction (IUGR) is a key developmental programming factor which might impair both the feed efficiency of lambs and meat quality, since it deeply impacts skeletal muscle and adipose tissue development. To determine the effect of birth weight on the growth performance, ruminal parameters, digestibility, non-carcass components, carcass traits, physicochemical characteristics and fatty acid profile of meat, two experimental groups (six animals in each group) of male Merino lambs with different body weights (BW) at birth (low BW (LW; 3.88 ± 0.281 kg) and high BW (HW; 5.80 ± 0.647 kg)) were used. The lambs were penned with their corresponding ewe during the natural suckling period, being weaned at 15 kg. Then, the lambs were penned individually and offered a complete pelleted diet during the fattening period. All the animals were slaughtered when they reached 27 kg of BW. After weaning, both daily dry matter intake (578 vs. 615 g/day; *p* = 0.021) and average daily gain (141 vs. 190 g/day; *p* = 0.004) were significantly lower in LW lambs, and a higher feed:gain ratio was recorded for this group (3.98 vs. 3.45; *p* = 0.008). Carcass traits did not show differences (*p* > 0.05) between both groups of lambs, except for higher chilling losses for the LW group (3.29 vs. 2.69%; *p* = 0.012). Additionally, higher amounts of kidney knob and channel fat were observed for LW lambs (85.4 vs. 152 g; *p* = 0.028). Apart from a higher hardness of meat in LW lambs (152 vs. 189 Newtons, *p* = 0.040), no other differences (*p* > 0.05) were observed in the physicochemical traits of this product; however, the meat of LW lambs tended (*p* = 0.057) to contain more total fatty acid content with a higher (*p* = 0.041) proportion of saturated fatty acids than the meat of HW lambs. In conclusion, under the conditions of the present study, a low body weight at birth increased the deposition of fat in carcass and non-carcass components during the fattening period of lambs, thus reducing animal performance and worsening the nutritional indexes of the meat. Accordingly, it seems reasonable to try to identify these animals during early life, to be sold as suckling lambs in the meat market instead of being fattened.

## 1. Introduction

Intrauterine growth restriction (IUGR) promotes low birth weight [1,2,3,4] mainly as a consequence of the malnutrition of the dam or placental insufficiency during the late pregnancy. Below the optimal weight range for lamb survival (4 to 6 kg), mortality is higher during the three first days of life [3,4]. Moreover, the surviving offspring obtained from these dams may present reduced daily weight gain [5,6], with other long-term effects such as a modified development of skeletal muscle and adipose tissue during the postnatal period. This is due to the fact that under inadequate maternal nutrition, skeletal muscle has a lower priority in nutrient partitioning compared with the brain and heart [7]. Under these circumstances, on the one hand, lower numbers of muscle fibers may be developed; on the other hand, the skeletal muscle cells may acquire insulin resistance during the fetal period to save glucose for other more demanding tissues, and these changes can persist later on during the lifespan [7]. Aligned with this statement, it must be mentioned that increased adiposity after a fattening period of early feed-restricted lambs (postnatal) is partially a consequence of insulin resistance patterns developed during early life, which may affect meat quality traits (e.g., more saturated intramuscular fat) [8]. These long-term effects of maternal nutrition (e.g., higher body fat deposition, low formation of lean muscle, obesity and metabolic syndrome) can be embraced under concepts such as the thrifty phenotype hypothesis, fetal programming (pre-natal feed restriction in utero) or developmental origins of health and disease (DOHaD, describing both pre- and post-natal feed restriction) [9,10,11,12]. Understanding how low body birth weight affects all these traits (body composition, carcass characteristics, organ development and meat quality (e.g., texture, fat deposition or fatty acid profile)) might help us to comprehend the reasons behind the high heterogeneity of fattened lambs in Mediterranean countries. This knowledge should be used, on the one hand, to implement strategies (e.g., management, selection, etc.) to keep consistent quality standards (e.g., tenderness, marbling, etc.) in the characteristics of a particular product (e.g., protected geographical indications), and on the other hand to increase the feed efficiency of the animals and the profitability of the farm. However, most of the studies found in the literature are focused on beef cattle [13,14], there being only limited evidence of the fetal programming effects on animal performance during the fattening period of lambs [10]. Therefore, further studies are required to understand the biological basis of this variability, and to quantify the repercussion on the economy of the farm. 

The present study was carried out to investigate the effects of a low birth weight of lambs on growth rate, ruminal parameters, digestibility, non-carcass components, carcass traits and meat quality (e.g., physicochemical characteristics, such as proximate analyses, fatty acid profile and texture) after a fattening period. Our initial hypothesis was that a low birth weight of lambs promotes long-term effects, thus affecting animal performance and meat quality traits during the fattening period.

## 2. Materials and Methods 

### 2.1. Animals and Diets

All handling practices involving animals followed the recommendations of Directive 2010/63/EU of the European Parliament, the Council on the protection of animals used for scientific purposes and the IGM-CSIC Animal Experimentation Committee (protocol number 2015-04). The multiparous Merino ewes of the experimental flock (90 animals) were treated with intravaginal progestagen sponges in order to synchronize the estrus; then they were allowed to carry out natural mating. All the ewes were fed according to the National Research Council guidelines [15] during gestation and postpartum periods. After lambing, twelve Merino lambs (singletons) were classified in two groups (*n* = 6 per group) on the basis of their live body weight (BW) at birth: high weight (HW; average BW 5.80 ± 0.20 kg) and low BW (LW; average BW 3.88 ± 0.08 kg). The male Merino lambs were penned individually (1.5 × 1.5 m covered with a sawdust bed) with their corresponding ewe during the suckling period for natural breeding. Each lamb was weaned progressively at a BW of 13.5 kg, restricting the suckling time to two hours (from 10:00 to 12:00 a.m.). The rest of the time the lambs were separated from the dams, and were allowed free access to a complete pelleted diet (CPD, formulated with 43% barley, 15% corn, 24% soybean, 15% barley straw, and 3% vitamin–mineral premix) and alfalfa until being 15 kg (BW). Then, each weaned lamb was penned individually and offered the same CPD (35 g/kg LBW per day, which supplies 2.5 Mcal ME/kg and 174 g of crude protein/kg DM). Detailed information on the ingredients and chemical composition of the CPD and procedures to obtain the analytical data can be found in Santos et al. [16]. Fresh drinking water was always available. All the lambs were weighed twice weekly before feeding throughout the whole experiment, and received the CPD once a day at 09:00 h; the daily amount of feed offered was adjusted twice a week on the basis of BW. Leftovers (if any) were collected daily, pooled in weekly composites for each animal, and analyzed for DM content. 

### 2.2. Determination of Dry Matter and Organic Matter Digestibility 

Digestibility was determined when each animal reached a BW of 20 kg using acid insoluble ash (AIA) as an internal marker. Briefly, feed and feces were sampled from each animal over 9 days. These samples were weighed and pooled in composite samples for each animal until AIA analysis following the procedures described by Santos et al. [16].

### 2.3. Slaughter Procedure, Ruminal Sampling and Carcass Characteristics

All the animals were slaughtered after a fattening period of at least 50 days with a target BW of 27 kg. Feed and water were withdrawn during 12 h, and each lamb was weighed (slaughter BW). Animals were then stunned, slaughtered by exsanguination from the jugular vein, eviscerated and skinned. The procedures to register the weight of the organs, fat depots, carcasses characteristics, commercial cuts and color parameters of subcutaneous fat in the lumbar region (L* a* b* coordinates (D65, 10°) measured using a Minolta CM-2002 chroma meter (Konica-Minolta Sensing, Inc., Langenhagen, Germany) can be found in Santos et al. [8,16]. Moreover, ruminal liquid was filtered and a 0.8 mL sample was added to 0.5 mL of deproteinizing solution (20 g metaphosphoric acid and 0.6 g crotonic acid/L in 0.5 N HCl). Then a Shimadzu GC 2010 gas chromatograph equipped with a column TR-FFAP 30 m × 0.53 mm × 1 µm (Supelco, Barcelona, Spain) was used to measure ruminal fermentation end-products (volatile fatty acids (VFAs)).

### 2.4. Physicochemical Evaluation of Meat

The values of pH were determined in the *longissimus thoracis* (LT) muscle (6th rib, on the right side of each carcass) at 0 h, 45 min and 24 h postmortem according to Blanco et al. [17]. The LT muscles were used to perform proximate chemical analyses and fatty acid profiling according to previous studies [18,19]. Briefly, lipids were extracted from 1 g of freeze-dried meat using a mixture of chloroform–methanol (1:1, *v*/*v*). Lipid aliquots (~10 mg) from each sample were methylated using sodium methoxide solution. For quantitative purposes, 1 mL of internal standard (1 mg/mL of 23:0 methyl ester) was added before methylation. The fatty acid methyl esters (FAMEs) were analyzed using a gas chromatograph with a flame ionization detector (Agilent Technologies, Model 7890A) equipped with a 100 m SP-2560 column (Supelco, Bellefonte, PA, USA), and setting a temperature program plateauing at 175 °C and a split ratio of 50:1. Hydrogen was used as a carrier gas with a flow rate of 1 mL/min, and the injector and detector temperatures were set at 250 °C. For the identification of the FAMEs, reference standards #463 and #603 obtained from Nu-Chek Prep Inc. (Elysian, MN, USA) were used. Other FAs, not included in the standard mixtures, were identified by their retention times and elution orders [19]. 

At 24 h postmortem both sides of the fresh *longissimus lumborum* (LL) were transversely cut into four different portions each side (2.5 cm thick), which were assigned to three storage times (no storage, 3 and 7 days; two slices per storage time, one used to measure texture and the other one to quantify lipid oxidation). The remaining two slices (one per side) were used to study the color evolution of meat. Except for day 0 portions, the other six slices obtained from each animal (3 and 7 days and color evolution) were placed in impermeable polypropylenes trays (one per animal) and wrapped with an oxygen-permeable polyvinylchloride film (oxygen permeability of 580 mL/m^2^ per h). The packaged meat was then stored under refrigerated conditions (3 ± 1 °C). Two slices (one per side) were used for the measurement of color on the cut surfaces at 24 h postmortem (day 0), and after 1, 3, 5 and 7 days of subsequent refrigerated storage [20]. Each sampling day the trays were unwrapped to measure lightness (L*), redness (a*), yellowness (b*), hue angle (h*) and chroma (C*) values (Centre Internationale de l’Eclairage, 1986) using a Minolta CM-2002 chroma meter (Konica-Minolta Sensing, Inc., Langenhagen, Germany) in the same two slices (one per side, duplicate measurement in each slice) every single day. These two slices were discarded after the last color measurement (7th day). 

The first day 0 portion of each animal was vacuum packaged and cooked in a water bath at 80 °C for 40 min, cooled in an ice bath for 30 min, stored overnight under refrigeration conditions, and then used for texture profile analysis (TPA) following the method described by Herrero et al. [21] with modifications. Briefly, three 1 cm^3^ meat cubes obtained from each portion were compressed twice (with the force parallel to the muscle fibers) to 80% of their initial height, using a cylindrical probe running at 0.5 mm/s and having an elapsed time between compressions of 5 s. The other two portions (days 3 and 7) in refrigerated trays were vacuum packaged, cooked, cooled, refrigerated, stored overnight and analyzed for TPA as described for day 0 portions. 

The second day 0 portion of each animal was stored at −20 °C to measure thiobarbituric acid-reactive substances (TBARS, [22]). Briefly, the raw meat samples were cut into 2.5 g pieces and homogenized for 30 s at 13 000 r.p.m. with 20 mL of distilled water using a T25 digital Ultraturrax (IKA, Staufen, Germany). Then, 5 mL of 25% trichloroacetic acid was added, centrifuged, filtered, and 3.5 mL was transferred to a 10 mL screw-cap tube with 1.5 mL thiobarbituric acid (0.6%). The samples were heated at 70 °C for 30 min and, after being cooled on ice for 10 min, the absorbance was measured at 532 nm. The results were expressed as mg MDA/g for meat, malondialdehyde (MDA) being the main product of lipid peroxidation. The other two portions (days 3 and 7) in the refrigerated trays were sampled and stored at −20°C for TBARS analyses, as described for day 0 portions. 

### 2.5. Calculations and Statistical Analysis

Average daily gain (ADG, g/day) was estimated as the regression coefficient (slope) of body weight against time whereas the feed to gain ratio or ‘feed conversion rate’ (FCR) was obtained by dividing the DMI per day by the previously estimated ADG during the fattening period (g DMI/g ADG). After assessing for normality by using the Saphiro–Wilk test, feed intake, growth performance, digestibility and ruminal fermentation data, together with organ weights and carcass characteristics and components, color of subcutaneous fat, chemical composition and fatty acid profiles were analyzed by one-way ANOVA using the GLM procedure of SAS [23], and considering the animal as the experimental unit. The changes in pH of LT after slaughter, TBARS, TPA and meat color during refrigerated storage were analyzed as repeated measurements by the MIXED procedure of SAS [23], using the animal nested to group as error to test the fixed effect of the experimental treatment. Storage time and the interaction between treatment and storage time were contrasted against the mean square of time × animal (treatment). Different covariance matrices were evaluated using the Schwarz’s Bayesian information criteria. The level of significance was determined at *p* < 0.05 and a trend towards significance was declared when *p* < 0.10. Means were separated using the least significant difference procedure.

## 3. Results

### 3.1. Animal Performance and Digestive Parameters

The growth performance of both groups of lambs is shown in Table 1. During both the suckling and fattening periods, the average daily gain was lower in LW lambs, probably due to a reduced daily DMI (not measured during the suckling period; 578 vs. 615 g/d in the fattening period; *p* = 0.021). ADG values (141 vs. 190 g/day; *p* = 0.004) were lower in LW lambs during the fattening period. As a consequence, LW lambs required more days than HW lambs (53 vs. 32 days, *p* < 0.01) to reach the weaning weight (15 kg), needed longer (*p* = 0.004) fattening period (88 days) than HW lambs (64 days) to reach the targeted slaughter BW (27 kg), and presented poorer feed:gain ratios during the last phase (3.98 vs. 3.45; *p* = 0.008). However, differences between HW and LW lambs were not observed for rumen fermentation parameters when slaughtered, but dry matter and organic matter digestibility tended (*p* < 0.10) to increase in LW lambs (Table 1).

### 3.2. Non-Carcass, Carcass and Meat Quality Traits

Table 2 summarizes the visceral organ mass of lambs at slaughter (27 kg). No differences between HW and LW lambs were observed for most visceral organ mass, except for lower weights in LW lambs for blood (1095 vs. 1221 g; *p* = 0.031) and small intestine (637 vs. 807 g; *p* = 0.004). On the contrary, the kidney knob and channel fat depot was heavier in the LW lambs (152 vs. 85.4 g; *p* = 0.028). It is worth mentioning that the digestive content tended to be lower in LW lambs (4.72 vs. 4.2 kg; *p* = 0.069), but a similar comparison between both groups was obtained when non-carcass components were expressed as the proportion of empty BW.

Table 3 shows the carcass characteristics of fattening lambs according to their birth weight ranges. No differences (*p* > 0.05) were observed between HW and LW lambs for hot and cold carcass weights or any of the carcass components. However, LW lambs showed a higher proportion of chilling losses (3.29 vs. 2.69%; *p* = 0.012), and increased subcutaneous fat lightness (L*, 72.1 vs. 68.5; *p* = 0.023) when compared to HW lambs. 

The change in muscle (LT) pH after slaughter and the color values of meat samples (LT) under refrigerated storage showed no differences (*p* > 0.05) for the birth weight of the animals studied (see Table 4). 

Regarding the proximate chemical composition of meat (Table 5), no significant differences were observed for any of the parameters studied (moisture, fat, ash, protein). Nevertheless, it is worth noting that numerically higher intramuscular fat values were obtained for the LW lambs (2.32 vs. 1.87 g/100 g of fresh matter; *p* = 0.107); probably the differences in this trait did not reach the significance level due to the low number of replicates in each group. Some difference between both groups of lambs were detected for the fatty acid (FA) profile of meat (Table 6). The total FA content of meat tended to be higher (*p* = 0.057) in LW lambs with greater amounts of both saturated (SFA) and monounsaturated FA (MUFA), whereas the polyunsaturated FA (PUFA) content was unaffected by birth BW. Differences in fatty acids resulted in a lower PUFA/SFA ratio in LW lambs (*p* = 0.022). 

The effect of birth weight on TBARS and the texture of the HW and LW cooked meats after the 0-, 3-, and 7-day ageing periods is shown in Table 7. Body weight at birth did not affect TBARS and showed a scarce effect on TPA characteristics, and the effect was not noticed in non-aged meat. Higher significant values for hardness in the LW meat samples (189 vs. 152 N; *p* = 0.040) were observed after 3 days of ageing. Lower springiness and chewiness for the LW group was observed after 7 days of ageing (0.491 vs. 0.427, *p* = 0.003; and 39.9 vs. 51.3 N, *p* = 0.012, respectively).

First category cuts (legs, ribs and foreribs); second category cuts (shoulders); third category cuts (breast, neck and tail).

## 4. Discussion

The present study was designed to study the long-term effects of low body weight at birth of lambs on growth performance, ruminal parameters, digestibility, non-carcass components, carcass traits, physicochemical characteristics and the fatty acid profile of meat after a fattening period. It is important to mention that the commercial conditions in the feedlot are far from the experimental controlled environment imposed in the present study (single housing in pens), where the movement and social interaction of the lambs was limited. These circumstances might have resulted in negative consequences on animal welfare [24], even though they could see, hear and touch the individuals of the next pens. Therefore, the results described previously might not be completely extrapolated to industrial conditions. Another limitation of the study is the small sample size; the statistical power of the trial was estimated for a few variables (e.g., ADG, FCR, DM digestibility, kidney knob and channel fat) according to the minimal difference and standard deviation expected [16]. The results highlighted that at least five to seven per treatment would be required to achieve a statistical power around 80% with the α risk of 0.05. The risk of error type II might be high for the other variables where the statistical power was not evaluated. Accordingly, *p*-values should be interpreted with caution and trends to significance must also be considered. Nevertheless, it must be pointed out that for most of the variables showing no significant differences between groups, the variation between means was less than 10%, which reduces the impact of type II error.

As expected, HW lambs showed higher ADG and lower feed to gain ratio, this being in accordance with predictable values [15,25]. In contrast, the LW lambs showed a lower daily DMI, which explains the poorer feed:gain ratio observed in these animals when they were slaughtered at the same body weight (27 kg). These differences were not related to variations either in the ruminal fermentation pattern or digestibility, whose values were similar to those reported for fattening lambs raised under similar conditions [26].

Daily dry matter intake might have been reduced in LW lambs due to the lower small intestine mass and a decreased surface area to effectively absorb nutrients from feed. This is probably the reason why LW lambs tended to increase the digestibility of feed in a failed attempt to compensate this physical limitation of the gut. Taken together, all these effects might explain, at least partially, the higher animal performance of HW lambs. Other studies in piglets with limited early growth [27], or lagomorphs undergoing feed restriction [28], on the other hand, have also demonstrated the low performance of these animals due to their inability to engage compensatory gain or compensatory feed intake, but higher masses for the small intestine of these animals were observed as well, probably as an adaptation mechanism to the increased absorption of nutrients. Therefore, the apparent lack of agreement among these studies and our results, as far as the small intestine is concerned, might be related to both the phase of feed restriction in each case [29], this being more critical in ruminants when it takes place at the mid- and late-gestation period [30], and the plasticity of this organ, which may allow compensation under several circumstances [29]. 

Apart from the smaller mass of the small intestine in the LW group, a second mechanism explaining the reduced daily weight gain of these animals might be related to the numerically higher values of intramuscular fat content and increased fat deposition in other depots of the carcass (e.g., kidney knob and channel fat) when compared to HW. This finding, which is in agreement with previous experiments studying feed efficiency in early feed-restricted lambs [8], seems to be aligned with programming events caused by feed restriction during the early life (e.g., downregulation of several mitochondrial genes impairing β-oxidation of fatty acids), which enable the increase in fat depots when the circumstances allow for fat accretion [8,31]. Other factors involved in a higher fat accretion of the LW lambs might be related to a reduced myogenesis during the prenatal life [32], or to a limited increase in the size of the myofibers during the postnatal growth due to a reduced feed intake [33]; both factors are important in explaining the increment of fat depots in LW lambs because in these animals more dietary energy might have been directed towards fat accumulation instead of protein accretion if the maximum fiber size was achieved in the muscle [34].

The total FA and the profile obtained from the meat samples seems to support the increased accumulation of fat in the adipocytes of several depots in LW lamb. In fact, there was a greater amount of saturated (SFA, *p* = 0.041) and a trend for monounsaturated fatty acids (MUFA, *p* = 0.071), whereas those mainly incorporated into membrane phospholipids (such as n−3 and n−6 PUFA) were unaffected by birth BW (*p* = 0.443). These changes, which are aligned with the accumulation of triglycerides in adipocytes of rats, have been attributed to the increased gene expression of lipogenic enzymes after chronic feed restriction/refeeding [35]. As a consequence of the increased amount of saturated fatty acids, the SFA/PUFA nutritional index of the meat from LW lambs was worse when compared to those of the HW group. 

Other meat quality traits related to sensory and nutritional attributes, such as color changes and TBARS progression during the refrigerated storage of raw meat, showed no significant differences between the experimental groups in the present study, and were comparable to those previously measured in other studies with fattening lambs [8]. This indicates that lipid and myoglobin oxidation followed a similar pattern in the meat of both lamb groups. The lack of differences in lipid oxidation is in concordance with the lack of differences in the PUFA content, which are the main substrates for lipid oxidation in meat. 

During dry ageing, muscle proteases tend to make meat tenderer; however, on the other hand, in small meat pieces the steady increase in dry matter caused by the loss of water by evaporation and an eventual drip loss would exert the contrary effect. In this context, Martínez-Cerezo et al. [36] reported that the TPA hardness of lamb meat loin portions decreased during the first days of ageing and then increased to values similar to or higher than non-aged meat. This pattern was also found for HW meat in the present study. However, differences in hardness between the two experimental groups were detected in 3-day aged meat, not at 24 h postmortem, and therefore should be attributed to factors related to the ageing process. In fact, the higher hardness in LW meat after 3 days of ageing when compared to HW meat would suggest a lower efficiency of meat proteolysis in LW meat. This effect would not be caused by the effect of differences in pH on the proteolytic activity since a similar pH was found in postmortem meat. However, it might be explained by an effect of muscle growth rate on muscle microstructure factors, such as cytoskeletal protein conformation or myowater distribution, which in turn would impact the proteolysis rate and tenderness [37]. A higher growth rate in beef has been associated with increased myofibril fragmentation and tenderness due to ageing, and is not related to the activity of the calpain system [38]. On the other hand, the lack of differences found in hardness after 7 days of ageing might be the result of a confounding effect of the ageing-related moisture loss on the above-mentioned association between growth rate and tenderness.

LW samples also showed lower elasticity (springiness) than HW samples at day 7 of ageing. This effect could also be related to differences between the experimental groups in the structure of myofibrillar proteins, since these are the main muscle structures responsible for elasticity [39]. There was also an effect on chewiness (51.3 vs. 39.0 Newtons for HW and LW; *p* = 0.012), which was due to the lower LW elasticity and cohesivity values used to calculate this textural trait.

Additionally, fat depots may show differences in growth, cellularity and metabolism [40]. This could explain the particularities observed in subcutaneous fat as far as color parameters are concerned. It is known that subcutaneous white adipose tissue is more prone to browning (brown adipocytes in white fat) than visceral fat [41]. Brown adipocytes are rich in mitochondria, and thus are related to thermogenesis (e.g., very important for neonate) along the whole life. Nevertheless, brown tissue may be reduced by undernutrition, thus limiting energy expenditure and favoring fat deposition, adaptations appropriate for feed restriction [42]. These effects seem to be in accordance with the results observed in the present study, where lower numerical hue angle values (0° is red; 90° is yellow) and significantly higher values for the L* parameter were measured in the subcutaneous fat of the LW lambs, thus suggesting a lower browning degree of this depot after the fattening period. Therefore, it is tempting to speculate that this mechanism might be another reason explaining differences in the thermogenesis, and hence the higher fat accumulation and lower feed efficiency traits (e.g., growth rates), of the LW lambs.

Finally, it is very important to highlight that, according to our previous studies, the postnatal nutritional events during early life also cause long-term effects on the feed efficiency during the fattening period [8]. Therefore, it is probable that the lambs of the present study were facing a multifaceted situation caused not only by the low birth weight, but also by lesser vigor at birth, lower colostrum intake, reduced passive transfer of immunity, and lower immune status. All these collateral factors might also help to explain the higher number of days used by the LW lambs to reach the weaning weight (Table 1). 

## 5. Conclusions 

Under the conditions of the present study, low body weight at birth reduces the animal growth during both suckling and fattening periods, and increases the carcass fat depots and the amount of saturated fatty acids of the intramuscular fat, thus worsening the nutritional indexes of the meat. Moreover, the texture of the meat is worse in low birth body weight lambs. According to these results, it seems reasonable to try to identify these animals during the suckling period in order to release them to the meat market during this phase, instead of being raised in fattening systems. 

## Figures and Tables

**Table 1:** Feed intake, growth performance, digestibility and ruminal fermentation parameters in lambs born with high (HW) or low weight (LW) during the fattening period.

	HW	LW	RSD	*p*-Value
DMI (g/d)	615	578	23.26	0.021
Total DMI (kg)	39.5	50.7	5.855	0.008
ADG ^1^ (g/day)				
Birth-weaning	253	187	33.9	0.007
Weaning-slaughter	190	141	22.8	0.004
Days				
At weaning (15 kg LBW)	31.5	52.8	9.44	0.003
Under fattening period (27 kg LBW)	64.3	87.7	10.8	0.004
Feed:gain ratio	3.45	3.98	0.280	0.008
Digestibility (%)				
Dry matter	70.2	72.4	2.01	0.091
Organic matter	72.2	74.3	1.98	0.073
Rumen pH and volatile fatty acids (VFA)				
pH	6.54	6.31	0.598	0.437
Total VFA (mmol/L)	55.8	59.6	29.6	0.825
VFA proportions (mmol/100 mmol)				
Acetate	49.2	49.6	3.37	0.841
Propionate	31.4	33.9	4.73	0.394
Butyrate	9.93	7.75	3.62	0.321
Valerate	3.49	3.31	0.801	0.698
Isovalerate	3.29	3.07	1.19	0.749
Isobutyrate	2.62	2.38	0.775	0.610

^1^ ADG: average daily gain; RSD: residual standard deviation.

**Table 2:** Slaughter LBW (kg), digestive content (kg) and visceral organ weights (g) and fat depots (g) in lambs born with high (HW) or low weight (LW).

	HW	LW	RSD	*p*-Value
Slaughter weight (kg)	27.5	26.8	0.885	0.226
Digestive content (kg)	4.73	4.20	0.446	0.069
Blood	1221	1095	87.7	0.031
Heart	181	174	22.8	0.592
Respiratory tract (pharynx, trachea, lungs)	516	482	85.8	0.511
Liver	565	546	48.2	0.521
Spleen	80	92	33.6	0.549
Rumen	721	768	77.8	0.316
Small intestine	807	637	78.1	0.004
Large intestine	386	368	44.2	0.504
Visceral fat depots				
Omental	124	162	49.8	0.215
Mesenteric	207	207	36.5	0.991
Kidney knob and channel fat	85.4	152	45.2	0.028

RSD: residual standard deviation.

**Table 3:** Carcass characteristics of fattening lambs born with high (HW) or low weight (LW).

	HW	LW	RSD	*p*-Value
Hot carcass weight (kg)	12.1	12.2	0.409	0.771
Cold carcass weight (kg)	11.8	11.9	0.528	0.591
Chilling losses (%)	2.69	3.29	0.338	0.012
Dressing (%)	42.9	44.4	3.77	0.113
Proportion of cuts (%)				
First category	60.9	62.2	1.16	0.103
Second category	20.3	20.0	0.937	0.542
Third category	18.7	17.8	1.28	0.273
Subcutaneous fat color				
L*	68.5	72.1	2.20	0.023
a*	2.51	3.01	0.820	0.331
b*	8.95	8.89	2.39	0.972
Hue angle	73.8	70.7	4.87	0.314
C*	10.4	9.44	1.82	0.429

RSD: residual standard deviation.

**Table 4:** Chemical composition (g/100 g fresh matter; *M. longissimus thoracis*) of meat in lambs born with high (HW) or low weight.

	HW	LW	RSD	*p*-Value
Water	76.8	76.9	1.64	0.861
Crude protein	19.2	19.9	2.20	0.594
Crude fat	1.84	2.32	0.475	0.107
Ash	1.47	1.50	0.229	0.832

RSD: residual standard deviation.

**Table 5:** Carcass pH at different times after slaughter and color parameters of meat samples (M. *longisimus lumborum*) after 0, 3 or 7 days of refrigerated storage (ageing) in lambs born with high (HW) or low weight (LW).

	HW	LW	RSD ^1^	RSD ^2^	*p*-Value		
					Group	Day	G*D
pH							
0 h	6.54	6.64					
45 min	6.23	6.11	0.247	0.180	0.474	0.001	0.045
24 h	5.57	5.75					
L*							
0 days	43.0	43.6					
3 days	44.8	43.8	2.795	1.974	0.792	0.117	0.403
7 days	43.4	43.0					
a*							
0 days	9.27	10.1					
3 days	10.4	11.3	1.951	1.314	0.233	0.0045	0.983
7 days	9.44	10.2					
b*							
0 days	4.51	4.41					
3 days	6.08	6.87	1.826	1.370	0.642	<0.001	0.544
7 days	6.27	6.45					
Hue angle							
0 days	26.2	23.6					
3 days	30.3	31.5	8.255	6.325	0.870	0.004	0.672
7 days	33.0	32.9					
C*							
0 days	10.4	11.1					
3 days	12.1	13.2	2.090	1.266	0.252	<0.001	0.587
7 days	11.5	11.9					

^1^ RSD: residual standard deviation to compare groups; ^2^ residual standard deviation to compare groups within days.

**Table 6:** Fatty acid (FA) profile (mg/100 g fresh matter) of meat (*M. longisimus thoracis*) from fattening in lambs born with high (HW) or low weight (LW).

	HW	LW	RSD	*p*-Value
Total FA	1409	1985	461.2	0.057
Saturated FA	580	886	225.2	0.041
14:0	40.0	49.3	16.82	0.912
16:0	321	476	122.4	0.054
18:0	186	317	80.64	0.018
Branched FA (BCFA)	19.7	28.7	6.36	0.035
Monounsaturated FA (MUFA)	569	788	187.7	0.071
cis-MUFA	517	694	168.1	0.090
9c-18:1	456	618	151.3	0.092
trans-MUFA	52.3	93.7	31.8	0.047
10t-18:1	39.7	78.9	30.0	0.047
11t-18:1	5.00	4.33	2.02	0.609
Polyunsaturated FA (PUFA)	203	219	35.5	0.443
n-3	13.4	14.6	3.73	0.606
n-6	185	200	31.7	0.432
Total CLA	7.78	11.2	4.43	0.216
9c,11t-18:2	4.25	3.55	2.09	0.578
Nutritional interesting indexes				
n-6/n-3	14.1	14.4	3.21	0.853
11t/10t	0.128	0.063	0.05	0.049
PUFA/SFA	0.355	0.250	0.0671	0.022

RSD: residual standard deviation.

**Table 7:** TBARS (µg MDA g^−1^) and texture profiles of meat samples (M. *longisimus lumborum*) after 0, 3 or 7 days of refrigerated storage (ageing) and subsequent cooking in lambs born with high (HW) or low weight (LW).

	HW	LW	RSD ^1^	RSD ^2^	*p*-Value		
					Group	Day	G*D
TBARS							
0 days	0.64	0.38					
3 days	1.09	0.94	1.087	1.037	0.516	0.002	0.952
7 days	1.88	1.55					
Hardness (N)							
0 days	178	179					
3 days	152 ^a^	189 ^b^	28.45	21.056	0.280	0.471	0.009
7 days	176	171					
Cohesiveness							
0 days	0.556	0.551					
3 days	0.526	0.521	0.058	0.0505	0.131	0.132	0.145
7 days	0.603	0.527					
Springiness							
0 days	0.475	0.455					
3 days	0.456	0.444	0.0419	0.0302	0.041	0.359	0.019
7 days	0.491	0.427					
Chewiness (N)							
0 days	47.1	44.9					
3 days	36.5	44.2	10.17	7.576	0.518	0.064	0.002
7 days	51.3 ^b^	39.0 ^a^					
Hardness (N)							
0 days	178	179					
3 days	152 ^a^	189 ^b^	28.45	21.056	0.280	0.471	0.009
7 days	176	171					

^1^ RSD: residual standard deviation to compare groups; ^2^ residual standard deviation to compare groups within days. ^a, b^ Different superscripts in the same line indicate statistical differences (*p* < 0.05) between groups.
